# Anthropometric assessment of children’s nutritional status: a new approach based on an adaptation of Waterlow’s classification

**DOI:** 10.1186/s12887-020-1940-6

**Published:** 2020-02-11

**Authors:** Haroldo da Silva Ferreira

**Affiliations:** 1 Faculty of Nutrition, Federal University of Alagoas (UFAL), Campus A.C. Simões, BR 104 Norte, Tabuleiro do Martins, 57072-970, Maceió, Alagoas Brazil; 2 Postgraduate Program in Health Sciences, Institute of Biological and Health Sciences/UFAL, Maceió, Alagoas Brasil

**Keywords:** Anthropometry, Nutrition assessment, Wasting, Stunting, Overweight, Concurrent wasting and stunting, Children

## Abstract

**Background:**

The methodology currently used for nutritional assessment of populations classifies children according to four conditions: eutrophy, wasting, stunting, and overweight. However, children can be stunted and wasted concomitantly. Similarly, they can be stunted and overweight. These conditions are associated with greater susceptibility to mortality or chronic diseases, respectively. This work presents an adaptation of Waterlow’s classification (AWC), which discriminates six nutritional conditions. Additionally, it provides a command routine in Stata, which processes the z-scores of the anthropometric indices height-for-age and weight-for-height and presents the respective prevalence of the nutritional conditions.

**Methods:**

Data from two household surveys were used to demonstrate the application of AWC, which were conducted in 1992 (*n* = 1229) and 2015 (*n* = 987), with probabilistic samples of children (< 5 years) in Alagoas, Northeast Brazil. AWC is based on a cross-classification scheme, involving the categories obtained with height-for-age (z < − 2; z ≥ − 2) and weight-for-height (z < − 2; − 2 to 2; z > 2).

**Results:**

The prevalence obtained with AWC in 1992 and 2015 was, respectively: eutrophy (71.0/80.2), stunting (20.8/2.7), wasting (0.8/2.1), concurrent stunting and wasting (0.5/0.0), overweight (4.8/14.4) and short stature with overweight (2.0/0.5). The prevalence of wasting, concurrent wasting and stunting, and for short stature with overweight was never higher than 2.3%. Possibly these values should be much higher in countries where there is a high prevalence of undernutrition. In total, 472 children had low height-for-age. By the usual anthropometric classification, they would be classified as chronic undernourished. However, 39 (8.3%) of them were also overweight and seven (1.5%) had concurrent stunting and wasting, a condition at extreme risk of mortality, which is perhaps the explanation for its low prevalence in cross-sectional studies.

**Conclusion:**

In addition to identifying wasted, stunted and overweight children, AWC also identified children with two other conditions, which are generally neglected in most nutritional surveys. Each of these nutritional conditions have different characteristics (aetiology, preventive, and therapeutic approach, damage to the patient’s health, and priority level in public policy). Such aspects justify their identification in the distinct scenarios where nutritional surveys are developed.

## Background

For the effectiveness of interventions designed to promote health through dietary care or food and nutrition policies, it is imperative to know the nutritional status of the individual or target population [1]. In this regard, although there are various others resources, such as biochemical, clinical, and dietary data analysis, anthropometry is the resource most widely used for nutritional assessment, since it is non-invasive, cheap and, above all, it presents quite satisfactory results [2].

In the anthropometric assessment of children’s nutritional status, the variables weight, height (or length), sex, and age are combined to form anthropometric indices. These are currently expressed as percentiles or z-scores units. The values obtained in the assessed subjects are compared with those produced from a healthy population, which constitute the anthropometric standards. The diagnosis is made, fundamentally, by finding measures that, being sufficiently far from measures of central tendency in the standard data, are unlikely to occur in healthy individuals. Currently, the anthropometric standard used to assess children under the age of five years old is the so-called WHO-2006 standard [3].

At the clinical level (assessment of an individual) it is necessary to investigate whether anthropometric measurements far from the median value of the reference data are due to genetic characteristics or whether it is due to nutritional disorders. Thus, other indicators should be investigated. However, at the epidemiological level (population-based assessments), the interpretation is based on the expected frequency of measures according to the statistical distribution in the data of the anthropometric standard. Using the z-score concept applied to the normal curve, and assuming the cutoff point of <− 2 or > 2 standard deviations (SD) to designate deficit or excess for a given anthropometric index, the percentage of individuals beyond these cutoff points in the anthropometric pattern is about 2.3%, respectively. The prevalence of anthropometric extremes (indicative of nutritional disorder) in the evaluated population is defined by the value that exceeds this percentage [4, 5]. That is, a prevalence of 2.3% below or above two standard deviations for any anthropometric index is considered “acceptable” since this is the frequency found in the reference population.

When the evaluation is performed in a population, there is a need to classify individuals according to different nutritional conditions. The first known system proposed for this purpose was the Gomez classification (1956) [6]. This classification, based on percentage adequacy compared to the reference median of the weight-for-age index, does not consider stature. Thus, this classification does not distinguish between chronic (stunting) and/or acute (wasting) processes, but only represents the weight deficit for the child’s age and gender. For this reason, another classification was proposed by Waterlow, who introduced the distinction between wasting, defined as low weight-for-height (WHZ), and stunting, defined as low height-for-age (HAZ) [7]. Additionally, the Waterlow classification allows identifying children who are wasted and stunted at the same time.

Since then, several other criteria have been proposed. Currently, the methodology that has been used in epidemiological studies is the one proposed by the World Health Organization (WHO) [4], based on the application of cutoff points, expressed in z-score units, to establish anthropometric extremes indicative of nutritional disorders: The WHO Global Database on Child Growth and Malnutrition uses a z-score cut-off point of < − 2 SD to classify low weight-for-age (underweight), low height-for-age (stunting) and low weight-for-height (wasting). The cut-off points of > 2 SD identify children with high weight-for-height (overweight).

However, this criterion, as well as Waterlow’s classification, does not allow the discrimination of specific conditions that are distinct. For example, when classifying a group of children with low height-for-age (stunting), among them there may be children who have, simultaneously, in relation to his/her respective weight-for-height, three different situations: a) low WHZ (concurrent stunting and wasting); b) normal WHZ (stunting only) and; c) high WHZ (short stature with concomitant overweight. In this particular case, the term short stature is more appropriate than stunting, to prevent confusion with the ongoing undernutrition process in stunting: Protein-energy malnutrition and overweight are two mutually exclusive conditions.

Concurrent stunting and wasting is a strong risk factor for child mortality [8, 9]. Stunting but with appropriate WHZ is assumed to be a metabolic adjustment to chronic undernutrition. In the third situation (short stature with overweight), the occurrence of overweight excludes the possibility of a diagnosis of current protein-caloric malnutrition, but, on the other hand, is associated with greater susceptibility to chronic diseases, especially in adulthood [10].

Many regions around the world experience the double burden of nutritional problems, which constitutes a threat to the children’s health of the world’s lowest socioeconomic populations. Thus, health professionals and public policy managers should be committed to actions that reduce the prevalence of undernutrition and, at the same time, prevent the rising incidence of obesity. This can be achieved through integrated programs that incorporate infectious disease control and promote healthy eating and physical activity [11]. Therefore, it is necessary that epidemiological surveys adopt strategies that can discriminate all possible nutritional conditions detectable by anthropometry. This information has important implications for the development of actions and public policies for prevention and control.

This work was carried out to outline an anthropometric classification system for nutritional status assessment, to be used in epidemiological studies involving children under five years old.

## Methods

To facilitate reporting, from now on, the proposed procedure will be referred to as “AWC” (adapted Waterlow classification). Data from two household surveys were used to illustrate its discriminatory potential. The surveys were conducted in 1992 (*n* = 1229) and 2015 (*n* = 987). In both, the sampling processes allowed the representative samples of the children under five years of age to be collected in the state of Alagoas, Northeast Brazil [12, 13].

In this population, cases of stunting and/or wasting are mostly of nutritional origin. Cases of other etiologies are rare and, when identified through the application of the referred morbidity questionnaire, were excluded from the analysis, so were the children with anatomical or pathological alterations compromising their anthropometric evaluations.

Due to the nutritional transition process, the prevalence of nutritional disorders found in these surveys was quite different. So, these studies were chosen because they allow that the classification obtained with the AWC can demonstrate its ability to discriminate the various ways in which the nutritional disorders resulting from protein-energy imbalance may appear in the population.

### 1992 Survey

To obtain a representative sample, the sampling process used a multiple (three-stage) design. The first stage selected the municipalities (counties) to be included in the study (*n* = 20); the second stage selected the census tracts in each municipality (*n* = 8); and the third stage chose a starting point from which 15 consecutive households were visited. Thus, 2400 homes were visited. By the end of the study, 1370 children were identified. This sample would allow obtaining estimates of the most common health problems and the health services coverage, with acceptable margins of error: 95% significance (1-α) and 80% study power (1- β). It was possible to obtain information on 1229 children, resulting in a loss rate of 9.1%. Detailed description of the sample design is available in another publication [13].

### 2015 Survey

The original project aimed to estimate the prevalence of food insecurity in families in the state of Alagoas, for which a sample of 3366 families was analysed [12]. For the present study, all children under five years living in the selected households were considered eligible. The sample calculation, carried out a posteriori, considered the following parameters: overweight prevalence of 9.7% [5]; a population of 328,000 children; margin of sample error of 2.5% and; 1.5 for correction of the effect of the complex design. For 95% CI, the minimum sample size would be 860 children, to which was added 10% to cover potential sample loss (closed houses, empty houses, and refusals), totalling a sample number of 946 children. The final sample analysed was slightly higher (*n* = 987). Calculations were performed by using the StatCalc tool from Epi-info, version 7.2.1.0 [14].. The sampling process was described in detail by Costa et al. [12]. Briefly, the procedure was as follows: 1st - 30 of the 102 municipalities in the state were selected by systematic sampling with probability proportional to the number of inhabitants; 2nd - four census tracts per municipality were chosen by simple draw, respecting the proportion between the urban and rural sectors; 3rd - one block in each of the census tracts was randomly selected, and; 4th - a household was randomly chosen in each block, from which, moving clockwise, 31 consecutive homes/families were visited.

### Data collection

In both surveys, data were obtained through home visits, during which an interview was conducted with the children’s mothers or guardians. Then the children’s anthropometric measures were obtained using regularly calibrated equipment. All procedures were previously tested in a pilot study. The field team was composed of trained and systematically supervised anthropometrists. In 1992, body weight was measured with a Salter type portable scale (CMS PBW-235; CMS Weighing Equipment, London, England), accurate to 100 g. In 2015 a digital scale (Charder® MS6121R, Taichung City, Taiwan) was used, with a capacity of 250 kg and a precision of 100 g. In both surveys, the scales were calibrated weekly against standard weight. Children older than 24 months had their stature measured in the standing position with a vertical stadiometer, while in children aged 24 months or less, the length was measured with the child in the supine position, using a horizontal anthropometric ruler. The equipment in all the surveys included a non-flexible measuring tape, accurate to 0.1 cm.

Socioeconomic and demographic data were obtained during the interview, using structured forms pre-tested in pilot study.

### Adapted Waterlow classification

The AWC is based on a cross-classification scheme, involving the categories obtained by applying the cutoff ±2 SD to the indices HAZ and WHZ. For this, the variables weight, height, age, and sex were processed in the Anthro 3.0 software (World Health Organization - WHO, Geneva, Switzerland) to obtain the z scores of the indices according to the WHO anthropometric standard [3].

In this work, the term height (vertical measurement of a person from head to foot) and length (longitudinal measure obtained in the supine position) will be used interchangeably with the same meaning, along with the term stature.

WHO defines overweight in children under five years of age as WHZ greater than two SD above WHO Child Growth Standards median, which is why this methodological criterion was chosen instead of body mass index-for-age [15]. The low WHZ (wasting) was defined by applying the cutoff < − 2 SD. Stunting was identified by HAZ < − 2 SD [16].

From the combination of the two categories obtained with HAZ (<− 2 and > 2 SD) and the three related to WHZ (< − 2, − 2 to 2 and > 2 SD), the children were classified according to six nutritional conditions, as shown in Table 1. For the established categories, the already traditional nomenclatures were used (eutrophy, wasting/acute undernutrition, stunting/chronic undernutrition, and overweight). For the case of the concurrent stunting and wasting the term decompensated chronic undernutrition was proposed; and for the occurrence of stunting plus overweight, the term short stature with overweight was used.

**Table 1:** Classification of nutritional status of children under five years of age according to the combination of height-for-age (HAZ) and weight-for-height (WHZ) indexes and respective categories (low, normal, or high) defined by applying the cutoff ±2 standard deviations (SD)

HAZ (SD)	WHZ (SD)		
	Normal (≥ −2 to ≤2) [10]	Low (< − 2) [20]	High (> 2) [30]
Normal (≥ − 2) **[0]**	**[10]** Eutrophy	**[20]** Wasting (Acute undernutrition)	**[30]** Overweight
Low (< − 2) **[1]**	**[11]** Stunting (Chronic undernutrition)	**[21]** Concurrent stunting and wasting (Decompensated chronic undernutrition)	**[31]** Stunted and overweight (Short stature with overweight)

[] The numbers inside the square brackets are the codes used in the elaboration of the command routine in the Stata software, aiming to process the data and to present the respective prevalence of the nutritional condition categories. If used outside the context of processing in Stata, they should be removed.

To facilitate the operationalisation of the classification, a command routine was elaborated in the Stata/SE 12.1 for Windows (StataCorp LP, College Station, TX, USA). So that it can work, it is necessary to have in the database the variables HAZ and WHZ (written exactly like this) in z scores. The user can copy and paste the routine into the Stata command area. After pressing enter, categorical variables will be generated from the continuous variables HAZ (low and normal) and WHZ (low, normal, and high), according to the established cutoff points. Then, another variable (called classification) will be generated, which will consist of the six nutritional conditions resulting from the combination of the indicators. Finally, the prevalence of these conditions will be presented. This routine is demonstrated below:

- gen whz_cat = WHZ
- recode whz_cat min/− 2.0001 = 20–2/2 = 10 2.0001/max = 30
- label define whz_cat 20 “Wasting” 10 “Normal” 30 “Overweight”
- label values whz_cat whz_cat
- gen haz_cat = HAZ
- recode haz_cat min/− 2.000001 = 1–2/max = 0
- label define haz_cat 1 “stunting” 0 “Normal”
- label values haz_cat haz_cat
- gen classification = haz_cat + whz_cat
- label define classification 10 “Eutrophic” 11 “Chronic undernutrition” 20 “Acute undernutrition” 21 “Decompensated chronic undernutrition” 30 “Overweight” 31 “ Short stature and Overweight “
- label values classification classification
- ta classification

As shown in Table 1, each of the six categories obtained was designated as an indicator of a nutritional condition: eutrophy, acute undernutrition, chronic undernutrition, decompensated chronic undernutrition, short stature with overweight and overweight.

### Ethical aspects

The projects for the 2015 survey (process # 010102/0355) were approved by the Research Ethics Committee of the Federal University of Alagoas. The use of the database of the 1992 study was carried out with the consent of the holder of the rights of this material (Prof. Dr. Cesar Gomes Victora, from the Federal University of Pelotas, RS, Brazil).

## Results

Considering the two surveys, 2216 children were examined (1992: *n* = 1229; 2015: *n* = 987). Table 2 shows the distribution of socioeconomic and demographic characteristics of the samples. The distribution according to sex and age group in 1992 was similar to that in 2015. From 1992 to 2015, there was a significant reduction (*p* < 0.05) in the proportion of residents in the rural area, households with four or more residents, mothers with more than two children, children with low (< 2500 g) or with high (≥4000 g) birth weight, mothers with low schooling, and adolescents mothers. On the other hand, there was a significant increase in the number of families that had a refrigerator at home. In Brazil, only extremely poor people do not have this equipment at home.

**Table 2:** Distribution of children under five years of age, according to categories of socioeconomic and demographic variables by survey year (1992 and 2015). Alagoas, Northeast Brazil

Variable / Category	Sample distribution (%)^a^		*P* value (χ^2^)
	1992 n (%)	2015 n (%)	
Sex			
Male	621 (50.5)	495 (50.2)	0.860 ^ns^
Female	608 (49.5)	492 (49.8)	
Age range (months)			
0–24	509 (41.4)	434 (44.0)	0.226 ^ns^
24.1–60	720 (58.6)	553 (56.0)	
Low Birth weight (< 2500 g)			
Yes	79 (9.8)	58 (6.2)	0.006*
No	729 (90.2)	871 (93.8)	
High Birth weight (≥ 4000 g)			
Yes	117 (14.5)	66 (7.1)	< 0.001*
No	691 (85.5)	863 (92.9)	
Mother with 3 or more children			
Yes	729 (60.8)	293 (30.5)	< 0.001*
No	470 (39.2)	667 (69.5)	
Low maternal schooling (years of study ≤4)			
Yes	873 (72.0)	185 (20.3)	< 0.001*
No	339 (28.0)	725 (79.7)	
Maternal age group (years)			
≤ 20.0	169 (14.1)	66 (7.2)	< 0.001*
20.1–39.9	915 (76.3)	752 (82.5)	
≥ 40.0	115 (9.6)	94 (10.3)	
Residence area			
Urban	626 (51.0)	731 (74.1)	< 0.001*
Rural	602 (49.0)	255 (25.9)	
Number of people in household			
≤ 4	345 (28.4)	573 (58.0)	< 0.001*
> 4	872 (71.6)	414 (42.0)	
There is refrigerator at home			
Yes	463 (37.7)	933 (94.9)	< 0.001*
No	765 (62.3)	50 (5.1)	

^ns^ Non-significant difference; * Statistically significant difference (*P* < 0.05), according to chi-square test

^a^ The 1992 and 2015 surveys included 1229 and 987 children, respectively. However, for some variables, it does not add up to the total number of individuals in each study because missing information for some of the children

The prevalences obtained by applying the AWC are presented in Table 3. In 1992, chronic undernutrition was the most prevalent problem (20.8%), which lost population relevance in 2015 (2.7%). On the other hand, overweight in 2015 reached three times the prevalence observed in 1992 (4.8% vs 14.4%), surpassing all forms of undernutrition in terms of epidemiological relevance. During the two analysed periods, the prevalence of acute undernutrition, decompensated chronic undernutrition, and short stature with overweight exhibited values never higher than 2.3%.

**Table 3:** Distribution of children under five years according to the six categories of the adapted Waterlow classification of nutritional status, based on data obtained from two surveys (1992–2015) conducted in the state of Alagoas, Northeast Brazil

Nutritional condition	Survey		Δ%	Total n (%)
	1992 n (%)	2015 n (%)		
Eutrophy	873 (71.0)	792 (80.2)	13.0	1665 (75.1)
Chronic undernutrition (stunting)	256 (20.8)	27 (2.7)	−87.0	283 (12.8)
Acute undernutrition (wasting)	10 (0.8)	21 (2.1)	162.5	31 (1.4)
Decompensated chronic undernutrition^a^	6 (0.5)	0 (0.0)	−100.0	6 (0.3)
Overweight	59 (4.8)	142 (14.4)	200.0	201 (9.1)
Short stature with overweight	25 (2.0)	5 (0.5)	−74.5	30 (1.4)
**Total**	**1229 (100.0)**	**987 (100.0)**		**2216 (100.0)**

^a^ Concurrently wasting and stunting

Table 4 shows that only overweight showed a significantly different prevalence according to gender, affecting more boys than girls. Although the prevalence of decompensated chronic undernutrition has affected twice as many girls as boys, the small number of cases impaired to reach conclusions.

**Table 4:** Anthropometric classification of the nutritional status (adapted Waterlow classification) of children under five years according to gender. Data obtained from a survey conducted in 1992 in the state of Alagoas (Northeast Brazil)

Nutritional condition	Gender		PR (CI 95%)	Total n (%)
	Male n (%)	Female n (%)		
Eutrophy	426 (68.6)	447 (73.5)	0.93 (0.87–1.00)	**873 (71.0)**
Chronic undernutrition (stunting)	134 (21.6)	122 (20.1)	1.08 (0.86–1.34)	**256 (20.8)**
Acute undernutrition (wasting)	5 (0.8)	5 (0.8)	0.98 (0.28–3.37)	**10 (0.8)**
Decompensated chronic undernutrition^a^	2 (0.3)	4 (0.7)	0.49 (0.10–2.66)	**6 (0.5)**
Overweight	42 (6.8)	17 (2.8)	2.42 (1.39–4.20)*	**59 (4.8)**
Short stature with overweight	12 (1.9)	13 (2.1)	0.90 (0.42–1.96)	**25 (2.0)**
**Total**	**621 (100.0)**	**608 (100.0)**	**–**	**1229 (100.0)**

PR (CI 95%): Prevalence ratio and respective 95% confidence interval, calculated by Poisson regression with robust variance adjustment.

* Statistically significant difference (*p* = 0.002), according to the chi-square test

About age ranges (Table 5), it was possible to note the following: a) Chronic undernutrition was significantly more prevalent in the age groups over 12 months; (b) Decompensated chronic undernutrition affected exclusively children under 24 months and; C) the prevalence of overweight decreased with increasing age, in a way that the prevalence among children older than 48 months was lower (*p* < 0.05) than that in younger age groups. In the other nutritional problems, there were no significant differences.

**Table 5:** Anthropometric classification of the nutritional status (adapted Waterlow classification) of children under five years according to age range. Data obtained from a survey conducted in 1992 in the state of Alagoas (Northeast Brazil)

Nutritional condition	Age range (months) n (%) PR (CI 95%)					Total
	0–12	12.1–24	24.1–36	36.1–48	48.1–60	
Eutrophic	248 (83.5) 1 (Ref.)	144 (67.9) * 0.81 (0.73–0.90)	155 (63.8) * 0.76 (0.69–0.85)	171 (68.4) * 0.82 (0.74–0.90)	155 (68.3) * 0.82 (0.74–0.90)	**873 (71.0)**
Chronic undernutrition (stunting)	16 (5.4) 1 (Ref.)	47 (22.2) * 4.12 (2.40–7.01)	68 (28.0) * 5.19 (3.09–8.72)	65 (26.0) * 4.83 (2.87–8.12)	60 (26.4) * 4.91 (2.91–8.28)	**256 (20.8)**
Acute undernutrition (wasting)	6 (2.0) 1 (Ref.)	2 (0.9) 0.47 (0.10–2.29)	0 (0.0) -	0 (0.0) -	2 (0.9) 0.44 (0.09–2.14)	**10 (0.8)**
Decompensated chronic undernutrition^a^	4 (1.4) 1 (Ref.)	2 (0.9) 0.70 (0.13–3.79)	0 (0.0) -	0 (0.0) -	0 (0.0) -	**6 (0.5)**
Overweight	20 (6.7) 1 (Ref.)	13 (6.1) 0.91 (0.46–1.79)	11 (4.5) 0.67 (0.33–1.38)	10 (4.0) 0.59 (0.28–1.25)	5 (2.2) * 0.33 (0.12–0.86)	**59 (4.8)**
Short stature with overweight	3 (1.0) 1 (Ref.)	4 (1.9) 1.87 (0.42–8.26)	9 (3.7) 3.67 (1.00–13.4)	4 (1.6) 1.58 (0.36–7.01)	5 (2.2) 2.18 (0.53–9.03)	**25 (2.0)**
**Total**	**297 (100.0)**	**212 (100.0)**	**243 (100.0)**	**250 (100.0)**	**227 (100.0)**	**1229 (100.0)**

PR (CI 95%): Prevalence ratio and respective 95% confidence interval, calculated by Poisson regression with robust variance adjustment.

* Difference statistically significant according to the 95% confidence interval

^a^ Concurrently wasting and stunting

**Fig. 1:**
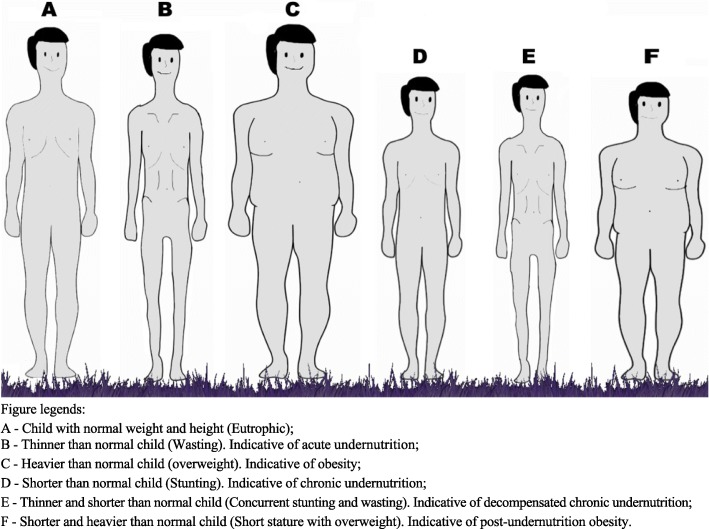
Representation of different combinations of stunting, wasting, and overweight in children of the same age and sex

Figure 1 is an illustration of children representing, respectively, the six possible anthropometric conditions that can be identified with the application of AWC.

## Discussion

In addition to identifying children with wasting, stunting and overweight, besides eutrophic children, the utilisation of AWC allowed the children’s identification with two other conditions, generally neglected in most nutritional surveys: those with short stature and overweight, and those with concurrent stunting and wasting, a condition that, due to its peculiar characteristics, we are suggesting designating it by the term decompensated chronic undernutrition.

Although in the data herein, the prevalence found for acute undernutrition, decompensated chronic undernutrition and for short stature with overweight was never higher than 2.3%, possibly these values should be much higher in countries where there are still high prevalences of stunting and wasting, such as Sudan (35 and 16%), Indonesia (36 and 13%), Nigeria (41 and 14%), Bangladesh (41 and 16%), Ethiopia (44 and 10%), Pakistan (44 and 15%) and Democratic Republic of the Congo (43 and 9%), respectively [17]. Taking the situation of the Democratic Republic of the Congo as an example, it may be asked: what percentage of stunted children are simultaneously wasted? This is important because this is a condition which increases about 12 times the chances of mortality in comparison to a child who is neither wasted nor stunted [9]. The under-five mortality rate in the Democratic Republic of the Congo in 2011 was 168 per 1000 live births. Most likely, many of these deaths involved children suffering from decompensated chronic undernutrition, but what was the contribution of this condition to the composition of this mortality rate? Would it be similar to the contribution attributed to other forms of undernutrition? Without a proper nutritional diagnosis, there is no answer to these questions.

The WHZ index allows to assess the adequacy of weight in relation to body height. Individuals with low WHZ are considered wasting, a condition indicative of acute undernutrition. This is because, especially in children, body weight can change rapidly [18].

In many epidemiological settings, the child survives in an unhealthy environment in many aspects. Among these, insufficient access to adequate food and successive cases of infection determine frequent cases of acute malnutrition. Consecutive acute cases thus increase the chances of mortality or, depending on the circumstances, determine adaptive processes that ensure the child’s survival in environments under such peculiarities.

In the data herein, the prevalence of low weight-for-height was always very low, unlike the height-for-age deficit, which was quite high in this first survey (1992). In cross-sectional studies, the prevalence of wasting is relatively lower than that of stunting [19], possibly, because of the high mortality in children with acute undernutrition [20] or because cases evolve to chronic condition represented by short stature (individuals either adapt or die).

The height-for-age index allows assessing the adequacy of linear growth in relation to respective age. As the height variable changes very slowly (compared to the variation in body weight), the observation of stature deficits can only be detected when nutritional deficiency has been established for relatively long periods. This is why short stature in children is an indicator of chronic undernutrition.

Among the effects of metabolic adjustments resulting from chronic exposure to inadequate diet and successive infections are included reduced growth velocity rate and, hence, lower metabolic rates and nutritional needs. In this case, the individual will evolve with short stature but will present a relatively preserved body mass in relation to the respective height. When not associated with wasting or with overweight, that is, short stature (stunting), but with appropriate weight to respective height, the condition is assumed as a metabolic adjustment to chronic undernutrition, allowing the individual to survive under conditions of nutritional deprivation.

Schoenbuchner et al. [21], through a retrospective cohort study based on a 40-year growth-monitoring records in rural clinics in Gambia, provided evidence that stunting is in part a biological response to previous wasting episodes. According to the authors, this finding suggests that stunting may represent an imperfect form of adaptation to more overt undernutrition (wasting) and that stunted children are not just short children but are children who earlier were more seriously undernourished and who are survivors of a composite process.

In this regard, it is important to consider some caveats to what has been called adaptation. According to Frisancho [22], adaptation is a process whereby the organism has attained a beneficial adjustment to the environment. When the responses to environmental stresses are not wholly successful, the term accommodation could be used because, even though they favour the survival of the individual, they also result in significant losses in some critical functions.

An article published by Martins et al. [23] summarises some of this damage: increased susceptibility to obesity, mostly abdominal obesity, lower fat oxidation, lower resting and postprandial energy expenditure, insulin resistance in adulthood, hypertension, dyslipidaemia and a reduced capacity for manual work, among other impairments. Thus, this process (short stature resulting from chronic malnutrition) will be more appropriately called accommodation rather than adaptation, as discussed by Frisancho [22].

Regardless, this process has limitations, so the children under such condition are already overwhelmed. Any additional disturbance or imbalance can lead to decompensation. Hence, the child who is short but of adequate weight-for-height may suffer a similar weight loss as that of the acute condition. Thus, the child will present a concurrent stunting and wasting picture (decompensated chronic undernutrition). Children in this category should receive maximum attention because their chances of mortality are extremely high [8].

Another situation arises from children who, having adjusted to an initial nutritional deficiency condition, cease to be exposed to such stress, achieving a better dietary pattern and/or being less exposed to infectious diseases. The metabolic changes resulting from this initial adjustment make these individuals more susceptible to obesity (and non-communicable chronic diseases in adulthood) [24]. The anthropometric profile of children in this condition is characterised by short stature associated with overweight. Popkin et al. [25] reported an association between stunting and overweight in children from Russia, Brazil, Republic of South Africa, and China, countries which are undergoing the nutrition transition process, suggesting that this increased susceptibility to overweight can be detected as early as childhood.

These findings evidence that chronic undernutrition may evolve to a decompensation or to overweight, according to prevailing environmental conditions (improvement or deterioration in dietary patterns and/or exposure to infections) as well as to individual biological characteristics. Gopalan [26] argues that the difference in the clinical picture reflects not a difference in the diet but a difference in the child’s capacity to adapt.

Of the total children analysed in this study, 472 children were identified with low height-for-age. By the usual anthropometric classification approach, they would be classified as chronic undernourished. However, 39 (8.3%) of them were also overweight. These children would then contribute to the occurrence of two errors: 1) overestimation of the prevalence of chronic undernutrition, as current overweight excludes the possibility of ongoing protein-energy malnutrition; 2) Since there is no cross-over of the anthropometric index data, these children would be double-counted, contributing to the prevalence of chronic malnutrition and also to overweight.

Also, there were seven (1.5%) children with concurrent stunting and wasting, which would lead to error in diagnosis and estimation of prevalence. However, from the point of view of health care, considering the high risk of mortality associated with this condition [8], the opportunity for an effective intervention to prevent such outcome would be missed. By the way, it cannot be said with certainty whether the low prevalence of wasting in contexts where the prevalence of stunting is high, is indeed a reality or is due to the high mortality that occurs among these individuals, a situation not detected in cross-sectional surveys.

Unfortunately, in this study, there was no way to present data on the relationship between malnutrition and mortality. However, in an ecological approach, considering the period between 1992 and 2015, when the prevalence of chronic undernutrition dropped from 20.8 to 2.7%, the child mortality rate in Alagoas fell from 88.7 per 1000 live births to 14.6 per 1000 live births [27]. It is very likely that the reduction in the number of malnourished children contributed to the observed decrease in the child mortality rate.

Lima et al. [28] investigated the temporal variation in the prevalence of undernutrition (height-for-age < − 2 z) in children under five years in Northeast of Brazil, in two successive periods (1986–1996 and 1996–2006). Additionally, they identified the main factors responsible for the observed changes. From 1986 to 1996, the prevalence of undernutrition fell from 33.9 to 22.2%, and between 1996 and 2006, from 22.2 to 5.9%. The authors concluded that with greater or lesser importance according to the period analysed, the factors related to the acceleration of the decline in undernutrition in Northeast Brazil were improvements in maternal schooling, in the coverage of water and sewage services, and especially the exceptional increase in family purchasing power.

Brazil has five macro-regions, among which the Northeast is the poorest of them. Alagoas is one of nine states located in this region. Therefore, it can be inferred that the data disclosed by Lima et al. [28] can be extrapolated to explain the observed decline in the prevalence of undernutrition herein reported. Similarly, the same factors would justify the reduction in the infant mortality rate in Alagoas.

Stunting, wasting, and concurrent stunting and wasting are distinct nutritional conditions that prevail within the same population. Thus, individuals in this population are exposed to several common risk factors [29]. However, risk factors and characteristics peculiar to each of these individuals contribute to the development of these different nutritional conditions, which require distinct attention from the health professionals, since it’s effects on the health, growth and development are variable, along with increased risk of morbidity and mortality. Therefore, it is necessary to establish priorities in the attention given to each condition.

Among the problems arising from poor feeding, obesity has been gaining prominence in terms of magnitude and health damage, and this is the reason why its prevention and control have received high priority in many countries.

Because of the high probability that obese children will become obese adults, these actions should be implemented as early as possible, i.e., since pregnancy, because maternal obesity is associated with fetal macrosomia (birth weight ≥ 4000 g), a risk factor for obesity [30]. Besides, eating habits are formed in the early years of life, so that the establishment of healthy habits in the early years tends to remain for the rest of their lives, promoting a body composition compatible with good health conditions [31].

Preventing childhood obesity means avoiding a broad spectrum of morbid conditions both during childhood as well as in later adult life. Especially in adulthood, excess body fat is a significant risk factor for chronic non-communicable diseases [32].

Regarding the AWC, two situations must be distinguished concerning overweight in children:

- Overweight children, but with normal height: Normal height suggests that the child did not experience substantial difficulties due to lack of access to food, as its growth occurred within the expected range. However, despite all the complexity surrounding the aetiology of obesity, this can be interpreted as resulting from a systematic positive energy balance.
- Short stature with overweight: As already mentioned, this child cannot have protein-energy malnutrition as he/she is overweight, which is indicative of positive energy balance, a situation incompatible with a current malnutrition framework. So, the short stature would be of constitutional origin or may be related to a prior undernutrition process, itself being the result of sparing metabolic adjustments of energy that, among other changes, reduce the growth rate. This economical metabolic profile makes the individual susceptible to obesity and its comorbidities and also makes the body weight control in these individuals much more difficult than those without low metabolic rate [23].

This study has some limitations:

- The AWC does not consider the weight-for-age index (WAZ). This index has limitations when used as an indicator of nutritional status because it is influenced by changes in both height and body mass. Thus, WAZ deficits or excesses may be due to HAZ or WHZ deficits or excesses, respectively, which makes the indicators based on this index unclear. However, it is important to note that weight-for-age becomes relevant as a monitoring criterion, i.e., when it comes to serial observations. Weight-for-age is also particularly useful in children under 12 months, not only because height measurement is not always accurately obtained in this age group, but above all because of the high speed at which weight evolves (or does not evolve during nutritional disorders) in the first year of life [33]. Because stature evolves more slowly, it requires relatively long periods of time before changes can be detected. Therefore, assessing weight in this age group is a more sensitive resource as a diagnostic criterion than indicators based on height.
- The AWC does not allow to discriminate the nutritional conditions according to their degree of severity. In this respect, it is argued that, as Waterlow [7] stated, “*to be useful, the classification needs to be simple*.” In this case, such stratification (mild, moderate and severe) could even be included within the framework of the proposed approach. However, the resultant situations would greatly hinder the feasibility of classification; in this case, more cumbersome than helpful: instead of six nutritional conditions, there would be 28 possibilities. However, when relevant, one or more of these six specific conditions can be individually explored about to their degree of severity. In this respect, it is important to emphasise that the classification system now proposed is suitable for population group assessment. For individual-level evaluation (in routine clinical practice or hospitalised children), there are already very well established proposals, such as the consensus published by the Academy of Nutrition and Dietetics and American Society for Parenteral and Enteral Nutrition [34, 35].
- AWC does not consider the presence of oedema, a key clinical sign of a severe form of protein-energy malnutrition, which is present in some nutritional classification systems, such as Wellcome Classification [36]. Bengoa [37], in 1970, analysed survey data from many countries considering as severe malnutrition all cases with oedema regardless of body weight. Oedema is a rare event and its identification is difficult to standardise in fieldwork, which hinders the inclusion of this criterion in household surveys.

## Conclusions

In addition to identifying wasting, stunting, and overweight children, the utilisation of the adapted Waterlow classification allowed the identification of children with two other conditions, generally neglected in most nutritional surveys. Each of these nutritional conditions has different characteristics, such as aetiology, preventive and therapeutic approach, damage to the patient’s health and priority levels in the public policies planning. Such aspects justify their identification and characterisation in the different scenarios where nutritional surveys are developed.

To allow comparison with previous studies that used different classification approach from the one proposed here, it is recommended to present the results, additionally, according to the traditional protocols: distribution of anthropometric indices (WAZ, HAZ and WHZ) according to the cutoff points proposed by WHO [15]. It is also important to consider all the other recommendations recently published by WHO/UNICEF [4].

## Data Availability

The datasets used and/or analysed during the current study are available from the corresponding author on reasonable request.

## Abbreviations

95% CI: 95% confidence interval
AWC: Adapted Waterlow classification
CNPq: Conselho Nacional de Desenvolvimento Científico e Tecnológico (National Council for Scientific and Technological Development)
FANUT: Faculdade de Nutrição (Faculty of Nutrition)
FAPEAL: Fundação de Amparo à Pesquisa do Estado de Alagoas (Foundation for Research Support of the State of Alagoas)
HAZ: Height-for-age index
PR: Prevalence ratio
SD: Standard deviation
UFAL: Universidade Federal de Alagoas (Federal University of Alagoas)
UNICEF: United Nations Children’s Fund
WHO: World Health Organization
WHZ: Weight-for-height
